# Metagenomic exploration of the virome of *Rhipicephalus sanguineus* ticks from Chachoengsao, Thailand

**DOI:** 10.3389/fmicb.2025.1736178

**Published:** 2026-01-07

**Authors:** Paola Mariela Saba Villarroel, Geraldine Piorkowski, Sedthapong Laojun, Florian Liégeois, Nuttamonpat Gumpangset, Dorothée Missé, Tanawat Chaiphongpachara, Sineewanlaya Wichit

**Affiliations:** 1 Department of Clinical Microbiology and Applied Technology, Faculty of Medical Technology, Mahidol University, Nakhon Pathom, Thailand; 2 Viral Vector Joint Unit and Joint Laboratory, Mahidol University, Nakhon Pathom, Thailand; 3 Unité des Virus Émergents (UVE: Aix Marseille Univ, IRD 190, INSERM 1207), Marseille, France; 4 Department of Public Health and Health Promotion, College of Allied Health Sciences, Suan Sunandha Rajabhat University, Samut Songkhram, Thailand; 5 MIVEGEC, Univ. Montpellier, CNRS, IRD, Montpellier, France; 6 Department of Medical Technology, LMI PRESTO, Faculty of Associated Medical Sciences, Chiang Mai University, Chiangmai, Thailand

**Keywords:** Bole tick virus 4, Brown dog tick phlebovirus 2, Changping tick virus 2, metagenomics, *Rhipicephalus sanguineus*, Thailand, viruses

## Abstract

Ticks are obligate blood-feeding ectoparasites that harbor a wide diversity of microorganisms. *Rhipicephalus sanguineus*, the brown dog tick, is globally distributed and poses significant veterinary and public health concerns due to its close association with companion animals and its occasional infestation of humans. However, the virome of this species in Thailand remains poorly characterized. In this study, we employed DNA Nanoball sequencing to investigate the virome of 80 *R. sanguineus* ticks, grouped into five pools, collected from dogs in Chachoengsao Province, Thailand, in 2023. Three viruses were identified: Brown dog tick phlebovirus 2 (BDTPV2), Changping tick virus 2 (CpTV-2), and Bole tick virus 4 (BLTV4), all detected in male ticks. These results highlight the need for further investigation into the ecological roles and biological significance of these viruses. Overall, our findings provide an updated perspective on the *R. sanguineus* virome in Thailand and underscore the importance of continued surveillance of tick-associated viruses within the One Health framework.

## Introduction

Ticks are obligate hematophagous ectoparasites and are considered the second most important vectors of human diseases worldwide, after mosquitoes. Taxonomically, they are classified into three families: Argasidae (soft ticks), Ixodidae (hard ticks, which account for more than 75% of described species), and Nuttalliellidae (represented by a single genus) (Souza et al., 2018; Maldonado-Ruiz, 2024; Fogaça et al., 2021). Ticks harbor a wide range of microorganisms, both pathogenic and non-pathogenic, collectively referred to as the tick microbiome (Wu-Chuang et al., 2021; Paez-Triana et al., 2023).

Most human tick-borne pathogens are transmitted by species of the family Ixodidae, particularly those belonging to the genera *Ixodes*, *Haemaphysalis*, *Hyalomma*, *Amblyomma*, *Dermacentor*, and *Rhipicephalus* (Souza et al., 2018). Within the latter genus, *Rhipicephalus sanguineus* (the brown dog tick) is the most widely distributed species globally. It is a three-host tick that primarily parasitizes dogs but can occasionally infest other hosts, including humans. Its close association with companion animals and ability to adapt to human dwellings increase the risk of pathogen transmission, raising significant veterinary and public health concerns (Saba Villarroel et al., 2024).

*R. sanguineus* is a recognized vector of multiple pathogens, including protozoa (e.g., *Babesia* spp., *Hepatozoon canis*), bacteria (e.g., *Anaplasma platys*, *Ehrlichia canis*, zoonotic *Rickettsia* spp., *Coxiella burnetii*) (Dantas-Torres et al., 2024; Sameroff et al., 2019), and highly pathogenic human viruses such as Crimean-Congo hemorrhagic fever virus (CCHFV) (Jafari et al., 2022; Gevorgyan et al., 2019; Shahid et al., 2021; Perveen and Khan, 2022), as well as, potentially, severe fever with thrombocytopenia syndrome virus (SFTSV) (Saba Villarroel et al., 2024).

Understanding the tick microbiome, however, requires a broader perspective than pathogen detection alone. Advances in next-generation sequencing (NGS) have enabled the high-throughput characterization of hundreds of other microbial taxa (Greay et al., 2018), including viruses belonging to diverse families such as *Nairoviridae*, *Phenuiviridae*, *Flaviviridae, Rhabdoviridae, Chuviridae, Reoviridae, Orthomyxoviridae,* and *Totiviridae* (Sameroff et al., 2019; Páez-Triana et al., 2025).

In Thailand, infestations of *R. sanguineus* among dogs are highly prevalent (Saba Villarroel et al., 2024). However, few studies have explored the virome of this species. One study, based on ticks collected in 2012 from Nan Province, identified two viruses: Changping tick virus 2 (family *Chuviridae*) and Bole tick virus 4 (unclassified family) (Temmam et al., 2019). Another study reported a partial genome of a *phlebovirus-like* virus from ticks collected in 2021 in Phra Nakhon Si Ayutthaya Province (Trinachartvanit et al., 2022).

Therefore, the present study characterizes the virome of *R. sanguineus* ticks collected in 2023 from dogs in Chachoengsao Province, Thailand, using DNA Nanoball sequencing. This work provides an updated understanding of the viral diversity and the evolutionary relationships of viruses associated with *R. sanguineus*.

## Methods

### Sample collection and extraction

In October 2023, ticks were collected from dogs in rural communities of Tha Takiap Subdistrict, Chachoengsao Province, in eastern Thailand, as previously described by Saba Villarroel et al. (2024). Each dog was thoroughly examined by hand, and all attached ticks were carefully removed while wearing disposable gloves to avoid direct contact. The specimens were placed in sterile containers and maintained alive for subsequent analyses. Live ticks were transported to the laboratory at the College of Allied Health Sciences, Suan Sunandha Rajabhat University. The ticks were washed with sterile phosphate-buffered saline (PBS), preserved in RNAlater, and identified as *Rhipicephalus sanguineus sensu lato* (s.l.) using established taxonomic keys (Farid, 1996). They were then categorized according to developmental stage and sex.

At the faculty of Medical Technology, Mahidol University, 50 pools of categorized *R. sanguineus* ticks were frozen in liquid nitrogen and crushed using a sterilized pestle. The homogenates were resuspended in sterile 1X phosphate-buffered saline (PBS). Prior to nucleic acid extraction, the samples were centrifuged at 8,000 rpm for 5 min at 4 °C, and the resulting supernatants were used for total DNA/RNA extraction using the Nucleospin RNA virus extraction kit (Macherey-Nagel, Germany), following the manufacturer’s instructions. The final elution volume was 80 μL, and the extracted samples were stored at −80 °C for subsequent molecular analyses.

### High-throughput sequencing

Five pools of ticks, comprising a total of 80 individuals, were selected for sequencing. Pool 1 included 18 adult males; Pool 2, four adult females; Pool 3, 12 adult males; Pool 4, two nymphs; and Pool 5, 44 adult males.

A total of 10 microliters (μL) of each nucleic acid extract was used for library preparation using the QIAseq FX Single Cell RNA Library Kit (Qiagen), following the manufacturer’s instructions (Gailey, 2023). Samples underwent random reverse transcription and complementary DNA (cDNA) synthesis, followed by amplification. The resulting double-stranded cDNA was enzymatically fragmented, and sequencing adapters and indexes were ligated to the fragments in accordance with the kit protocol. The prepared libraries were purified and quantified using the Qubit® dsDNA High Sensitivity (HS) Assay Kit and a Qubit 2.0 fluorometer (Thermo Fisher Scientific, USA) (Caceres, 2023).

High-throughput sequencing was carried out on the DNBSeq-G400 platform (MGI Tech, Hong Kong) employing DNA Nanoball (DNB) sequencing technology based on rolling circle amplification (RCA), which enables highly dense and accurate cluster generation without bridge PCR (Ruppeka Rupeika et al., 2024). Sequencing was conducted in a paired-end configuration, generating 2 × 150 base pair (bp) reads.

Raw paired-end reads were imported into CLC Genomics Workbench version 22.0.1 (Qiagen) for bioinformatic processing using default parameters.

Reads were then quality-filtered and trimmed, removing reads with a quality score below 0.99 and a length of less than 60 bp. Additionally, the first 20 nucleotides and the last 20 nucleotides were trimmed to eliminate potential sequencing artifacts, low-quality regions, and residual adapters. Host-derived sequences were removed by mapping reads against reference sequences and filtering out aligned reads. High-quality, non-host reads were subject to *de novo* assembly using CLC Genomics Workbench with default parameters, resulting in a set of contigs for each pool.

These contigs were compared against the NCBI database using BLASTn to identify the most similar reference sequences. Based on the best matches, reads were mapped back to the selected reference sequences to generate consensus sequences. To minimize reference bias, each consensus sequence was subsequently compared to its corresponding de novo assembled contig.

In parallel, all paired-end reads were independently analyzed using Kaiju software, which performs protein-level classification to accurately identify viral sequences within metagenomic datasets (Menzel et al., 2016).

### Ethical approval

The experimental protocol and tick sample collection were approved by the Institutional Animal Care and Use Committee of Suan Sunandha Rajabhat University (No. IACUC 66–002/2023).

### Phylogenetic analysis

The sequences obtained in this study were aligned with published sequences retrieved from the GenBank database using MAFFT version 7.526^1^, employing the FFT-NS-i method (Katoh et al., 2005). Potential recombinant sequences were identified using the Recombination Detection Program (RDP) version 5 (Martin et al., 2021) and removed from the dataset, except for Bole Tick Virus 4. For primary screening, the RDP, GENECONV, and MAXCHI methods were applied, while BOOTSCAN and SISCAN were used to confirm recombination signals (Martin et al., 2015; Martin et al., 2005; Padidam et al., 1999; Smith, 1992; Gibbs et al., 2000). Potential recombination events were also visualized using SplitsTree (version 3) through the construction of phylogenetic networks (Huson and Bryant, 2024). The resulting alignments were used to construct maximum-likelihood phylogenetic trees with IQ-TREE version 1.6.12 (Nguyen et al., 2015; Minh et al., 2020; Hoang et al., 2018). The best-fit nucleotide substitution model for each dataset was determined automatically using ModelFinder, integrated within IQ-TREE. Branch support was assessed using the ultrafast bootstrap approximation (UFBoot2) with 1,000 replicates (Kalyaanamoorthy et al., 2017).

The final phylogenetic trees were visualized, annotated, and edited using iTOL v7^2^, providing clear depictions of viral relationships and clade support.

### Accession numbers

The genome sequences generated in this study have been deposited in GenBank under the accession numbers PX454767–PX454770.

The raw sequencing data generated in this study have been deposited in the NCBI Sequence Read Archive (SRA) under BioProject ID PRJNA1371702.

The host reference genome used was *Rhipicephalus sanguineus* (GCF_013339695.2_BIME_Rsan_1.4_genomic).

## Results

Metagenomic sequencing of five pools of *Rhipicephalus sanguineus* generated between 85 and 273 million raw reads per pool. Sample 1 produced 259,061,882 raw reads, of which 253,353,346 remained after trimming, leaving 4,262,852 non-host reads (1.6%). Sample 2 (adult females) generated the highest number of reads, with 273,131,590 raw reads, 134,050,201 after trimming, and 18,371,805 non-host reads (6.7%). Sample 3 produced 85,120,864 raw reads, 41,195,609 after trimming, and 4,813,354 non-host reads (5.7%). Sample 4 yielded 110,148,716 raw reads, 53,078,522 after trimming, and 3,746,248 non-host reads (3.4%), while Sample 5 generated 118,989,850 raw reads, 56,597,673 after trimming, and 3,454,988 non-host reads (2.9%) (Supplementary Table 1).

### Viral diversity in *Rhipicephalus sanguineus* ticks

The detected viruses included Brown dog tick phlebovirus 2 (BDTPV2), Changping tick virus 2 (CpTV-2), and Bole tick virus 4 (BLTV4), all of which were identified in adult male ticks.

### Brown dog tick phlebovirus 2

Brown dog tick phlebovirus 2 (BDTPV2), first identified in *Rhipicephalus sanguineus* ticks from Trinidad and Tobago, is a recently described, unclassified member of the genus *Phlebovirus*, family *Phenuiviridae*, order *Hareavirales*. Unlike typical phleboviruses, which possess a tripartite, negative-sense single-stranded RNA (ssRNA) genome (L, M, and S segments), BDTPV2 is bi-segmented, lacking the M segment that encodes the glycoproteins required for host cell entry. The L and S segments encode the RNA-dependent RNA polymerase (RdRp) and nucleoprotein (NP), respectively (Sameroff et al., 2019; Bratuleanu et al., 2022).

**Figure 1:**
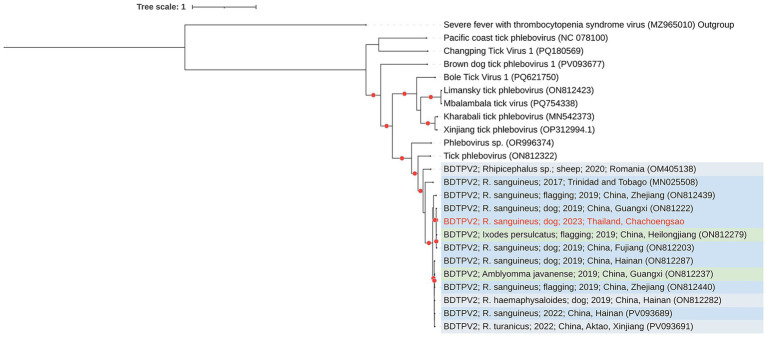
Maximum-likelihood phylogeny of the RNA-dependent RNA polymerase (RdRp) of Brown dog tick phlebovirus 2 (BDTPV2) from Chachoengsao Province, Thailand (2023), constructed alongside all publicly available complete BDTPV2 RdRp sequences in GenBank (as of 21 September 2025) and closely related unclassified phleboviruses. Sequences generated in this study are highlighted in red, and red dots indicate bootstrap support >95%. The tree was midpoint-rooted. Phylogenetic inference was performed using IQ-TREE2 with ultrafast bootstrap approximation (1,000 replicates) under the Generalized Time Reversible (GTR) substitution model.

In this study, BDTPV2 was detected in three pools of adult male ticks (pool 1, 3, and 5), and two complete L segments of BDTPV2 were recovered, designated BDTPV2-Thailand01 (pool 1, GenBank accession number: PX454767) and BDTPV2-Thailand02 (pool 5, accession number PX454768). Phylogenetic analysis of the RdRp amino acid sequences placed the Chachoengsao, Thailand genomes within a well-supported clade (bootstrap support >95%) alongside BDTPV2 strains previously reported from *R. sanguineus* ticks in Guangxi Province, China (ON812222), collected from domestic dogs in 2019 (Figure 1). The genome lengths of our sequences were 6,493 nucleotides, sharing >99% amino acid identity in the RdRp and exhibiting 100% query coverage relative to the Guangxi strain.

### Changping tick virus 2

Changping tick virus 2 (CpTV-2) belongs to the species *Mivirus changpingense*, genus *Mivirus* within the family *Chuviridae* (order *Jingchuvirales*). Its genome is circular, non-segmented, and composed of negative-sense single-stranded RNA (−ssRNA). The genome typically encodes three major proteins: the nucleoprotein (N), the glycoprotein (G), and a large (L) protein that contains the RNA-dependent RNA polymerase (RdRp) domain (Temmam et al., 2019; Maqbool et al., 2022).

**Figure 2:**
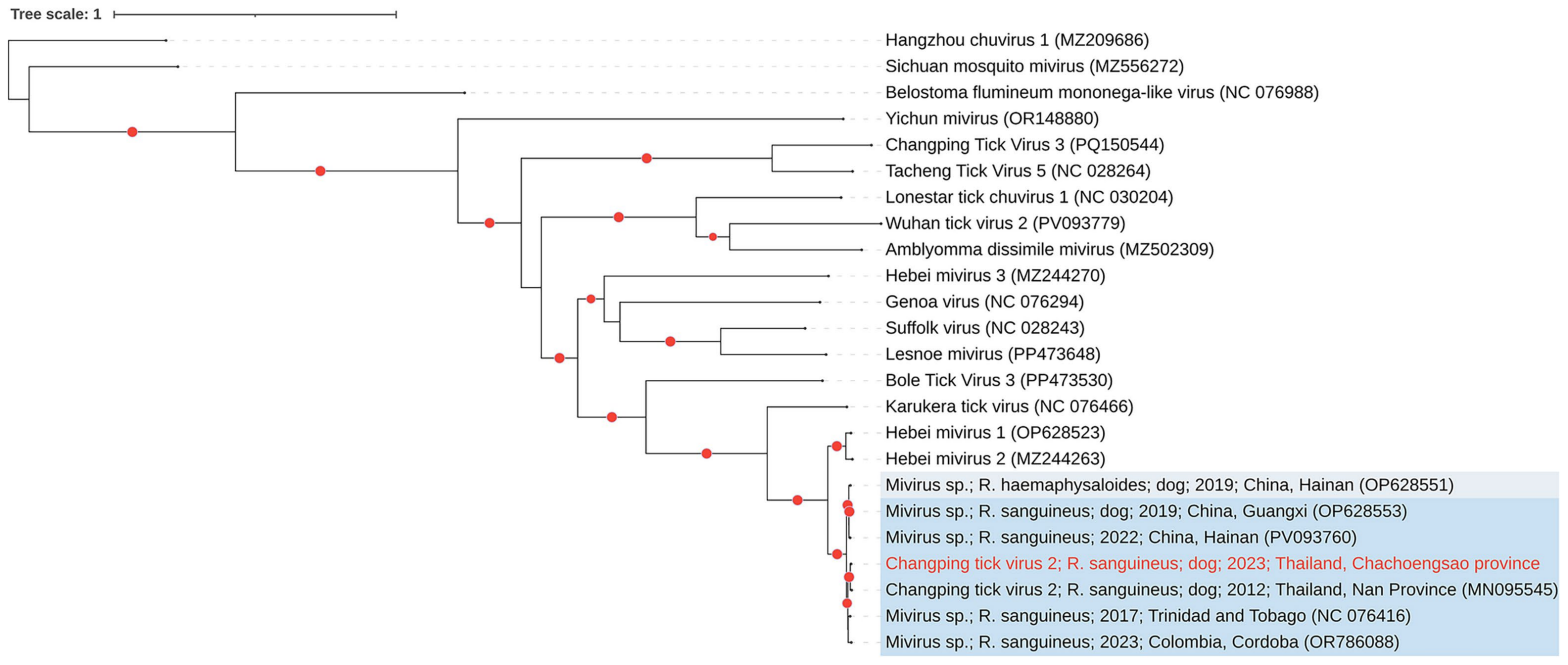
Maximum-likelihood phylogeny of Changping tick virus 2 (CpTV-2) from Chachoengsao Province, Thailand (2023), reconstructed from the complete genome together with closely complete related mivirus sequences available in GenBank (as of 21 September 2025). The sequence generated in this study is highlighted in red, and red dots indicate bootstrap support >95%. Phylogenetic inference was performed using IQ-TREE2 with the ultrafast bootstrap approximation (1,000 replicates) under the generalized time reversible (GTR) substitution model.

In this study, a CpTV-2 complete genome of 10,025 nucleotides was recovered from adult male tick pool 3 and designated CpTV-2 (GenBank accession number PX454770). Phylogenetic analysis placed this genome within a well-supported clade (bootstrap support >95%) alongside a CpTV-2 sequence previously reported from *R. sanguineus* ticks collected from dogs in Nan Province, Thailand, in 2012 (MN095545) (Figure 2), sharing >99% amino acid identity and 100% query coverage.

### Bole tick virus 4

Bole tick virus 4 (BLTV-4) is a positive-sense single-stranded RNA (ssRNA) virus whose genome organization resembles that of flaviviruses but remains unclassified, belonging to a growing group of unclassified positive-sense ssRNA viruses (Zhang et al., 2021). It was first detected in *Hyalomma asiaticum* ticks in China (Temmam et al., 2019).

**Figure 3:**
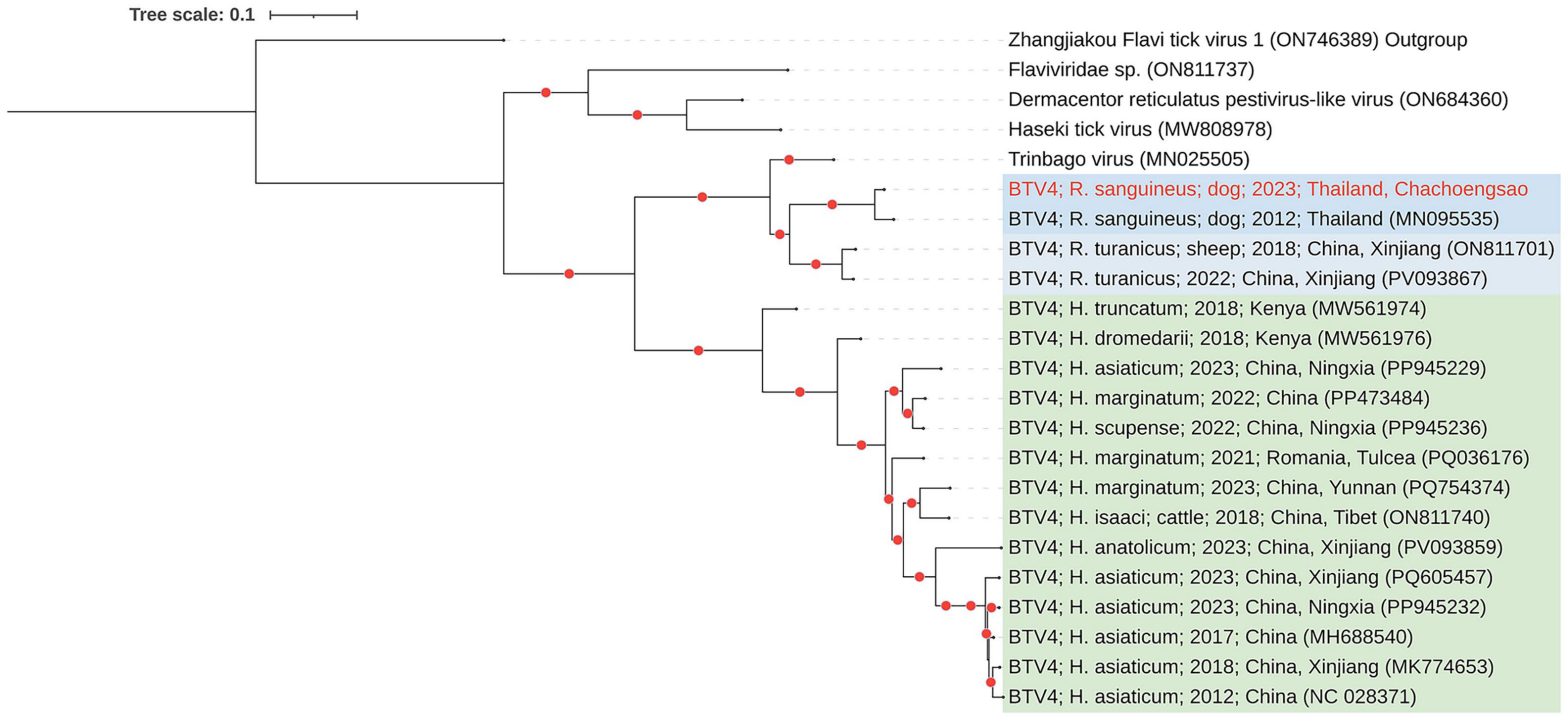
Maximum-likelihood phylogeny of the complete polyprotein of Bole tick virus 4 (BLTV4) from Chachoengsao Province, Thailand (2023), constructed together with all publicly available complete BLTV4 polyprotein sequences in GenBank (as of 21 September 2025) from *Rhipicephalus* and *Hyalomma* species, as well as closely related positive-sense single-stranded RNA viruses. The tree was midpoint-rooted. The sequence generated in this study is highlighted in red, and red dots indicate bootstrap support >95%. Phylogenetic inference was performed using IQ-TREE2 with ultrafast bootstrap approximation (1,000 replicates) under the Generalized time reversible (GTR) substitution model.

In this study, a complete 16,257-nucleotide genome was recovered from male tick pool 1 (GenBank accession number PX454769). Phylogenetic analysis places this genome within a well-supported clade (bootstrap support >95%) alongside a BLTV4 sequence from *R. sanguineus* ticks collected from dogs in Nan Province, Thailand, in 2012 (accession no. MN095535) (Figure 3), showing 95% query coverage and >98% nucleotide identity with this strain.

## Discussion

In this study, we employed DNA nanoball sequencing to characterize the virome of *Rhipicephalus sanguineus* (s.l.) ticks collected from dogs in rural communities of Tha Takiap Subdistrict, Chachoengsao Province, Thailand, in 2023.

Three recently described viruses were identified. The first, Brown dog tick phlebovirus 2 (BDTPV2), an unclassified phlebovirus, was found in all three male tick pools. This virus has been primarily detected in *Rhipicephalus* spp. (Sameroff et al., 2019; Wang et al., 2025) and in various regions, including Trinidad and Tobago (Sameroff et al., 2019), India (Desingu et al., 2025), Romania (Bratuleanu et al., 2022), Mexico (Laredo-Tiscareño et al., 2025), and China (Guo et al., 2022), suggesting that BDTPV2 is a widespread and stable component of the *Rhipicephalus* tick virome. In Thailand, a partial 490-bp RdRp sequence of a *Phlebovirus*-like virus related to BDTPV2 was also reported in 2021 in the central part of the country (Trinachartvanit et al., 2022), indicating that this virus has been circulating locally since at least 2021, thereby confirming its presence in Southeast Asia. Phylogenetic analysis revealed that our sequences were closely related to BDTPV2 sequences from Guangxi, China, a region bordering Vietnam. This highlights regional connectivity and underscores the potential for transboundary viral movement facilitated by their vertebrate hosts.

BDTPV2 is unlikely to pose a direct risk to vertebrate hosts, as it lacks the M segment required for host cell entry; alternatively, it may be a helper-dependent virus requiring assistance from another microbe or from the host itself to gain cellular entry (Sameroff et al., 2019), which could potentially pose a risk to vertebrates.

The second virus identified was Changping tick virus 2 (CpTV-2) (family Chuviridae). Phylogenetic inference showed that CpTV-2 clustered with *R. sanguineus* strains collected in Nan Province, Thailand, in 2012 (Temmam et al., 2019), located near the Laos border, approximately 800 km from our sampling sites, forming a strongly supported clade (>95% bootstrap support). This close relationship suggests conserved genetic features and relative evolutionary stability over the past decade.

Related sequences of *Mivirus* sp. have been increasingly discovered in ticks across different geographic regions, including China (Ni et al., 2023), Trinidad and Tobago (Sameroff et al., 2019), Mexico (Laredo-Tiscareño et al., 2025), and Colombia (Páez-Triana et al., 2025). Interestingly, many of these miviruses have been exclusively detected in *Rhipicephalus* spp., hinting at a possible adaptation to this tick genus. This virus remains poorly understood, and no vertebrate infections are currently confirmed.

The third virus identified was Bole tick virus 4 (BLTV4), an unclassified positive-sense ssRNA virus. BLTV4 has been reported in multiple countries and is primarily associated with *Hyalomma*, *Rhipicephalus* and *Dermacentor* tick species (Wang et al., 2025). Phylogenetic analysis places the sequences from this study within a well-supported clade alongside strains from Nan Province (bootstrap >95%), consistent with long-term circulation in regional tick populations. Sequences of BLTV4 recovered from *Rhipicephalus* spp. form a clade distinct from other species, displaying a clear clustering pattern according to tick genus. This supports the notion that viruses trajectories, (Wang et al., 2025).

Although phylogenetic trees constructed from different genomic regions showed broadly consistent topologies, recombination analyses using RDP5 and reticulate network visualization in SplitsTree revealed significant evidence of recombination in BLTV4 (Supplementary Figure 1). These findings suggest that it may undergo recombination under natural conditions, potentially contributing to its evolutionary dynamics. While the biological significance of this recombination remains unclear, such events can generate genetic diversity that may influence viral fitness, host adaptation, or interactions with other microorganisms within ticks.

Finally, BLTV4 is thought to be acquired through exposure to the blood of infected animals, and it may represent a potential viral pathogen, as suggested by studies of *Hyalomma* ticks collected from camels in Kenya (Zhang et al., 2021). Nonetheless, further research is needed to clarify its specific functions and pathogenic potential.

These viruses were detected in male ticks, a pattern that may be explained by their feeding behavior. Male ticks can take multiple blood meals, and studies have shown that a male previously attached to one dog can detach, move to a co-housed dog, and feed again. Additionally, males may remain on a host for extended periods, increasing their likelihood of acquiring microorganisms, including viruses (Dantas-Torres, 2010). In contrast, the absence of these viruses in nymphs and female ticks may be influenced by the higher abundance of endosymbionts (e.g., *Coxiella*-like endosymbionts), which encode essential pathways for the synthesis of B vitamins and their cofactors (Bonnet et al., 2017) and are believed to mediate microbial interference (Fogaça et al., 2021). Notably, although these viruses were mainly detected in adult male ticks in this study, the previously mentioned survey in Thailand reported a broader distribution, including nymphs and females (Temmam et al., 2019). This discrepancy highlights the need for further studies to elucidate the ecological role of these viruses within ticks, as well as the mechanisms underlying their acquisition and maintenance across different life stages and sexes.

Our study has several limitations. First, we analyzed a relatively small number of ticks, collected through opportunistic sampling from a single region in Thailand, which may limit the generalizability of our findings to other geographic areas or tick populations. Second, we did not examine tick eggs, preventing assessment of vertical transmission or the potential persistence of microorganisms across tick generations, and the number of males exceeded that of females and nymphs. Third, although short-reads sequencing provides sufficient resolution to assemble viral genomes *de novo* when combined with high coverage, the use of long reads sequencing could further improve the quality of metagenomic assemblies (Chen et al., 2022; Greenman et al., 2024). Despite these limitations, a key strength of this study is the comparison of newly collected ticks with historical data from the 2012 Nan Province study, providing valuable insights into temporal changes and trends in the tick virome. Moreover, our ticks were collected from a different geographic region, offering new regional insights; we analyzed a larger number of samples, increasing the robustness and representativeness of the findings; and we recovered complete genomes of BDTPV2, which were not reported in the samples collected in 2012 and were frequently detected in our dataset. Together, these advances expand the current understanding of tick-associated viral diversity and distribution in Thailand. Further large-scale studies are needed for a more comprehensive understanding of the tick virome, particularly in underexplored regions and host species.

Our findings have important One Health implications, as *R. sanguineus* ticks are closely associated with domestic dogs that often live near humans and livestock. The presence of diverse tick-associated viruses highlights the risk of cross-species viral transmission. Although none of the viruses identified in this study are currently confirmed as zoonotic, their presence emphasizes the importance of integrated surveillance to detect emerging threats and guide strategies to prevent potential spillover events.

## Conclusion

Climate change and environmental alterations are reshaping the distribution and abundance of ticks and other vectors, potentially expanding viral reservoirs and increasing the risk of emergence or re-emergence of vector-borne pathogens. The application of metagenomics has greatly enhanced our understanding of the microbiome of diverse vectors. In this study, we provide an updated characterization of the virome of *Rhipicephalus sanguineus* in Thailand, revealing the presence of three main viruses, two previously reported in 2012 and one more recently identified. Although these viruses are not currently known to pose a direct risk to humans or animals, ongoing surveillance of hidden viral populations is essential to better understand virus-vector interactions.

## Data Availability

The datasets presented in this study can be found in online repositories. The names of the repository/repositories and accession number(s) can be found in the article/Supplementary material.

## Footnotes

1
https://mafft.cbrc.jp/alignment/server/index.html

2
https://itol.embl.de/
