# Supplementary material for: Metagenomic exploration of the virome of Rhipicephalus sanguineus ticks from Chachoengsao, Thailand
Source: Front Microbiol. 2026 Jan 7;16:1736178. doi: 10.3389/fmicb.2025.1736178 (PMC12819733; doi:10.3389/fmicb.2025.1736178)
Supplement: Supplementary file 1 [file Data_Sheet_1.PDF]

Supplementary Material

Metagenomic exploration of the virome of *Rhipicephalus sanguineus* ticks from Chachoengsao, Thailand

Supplementary table 1. Summary of sequencing read processing per sample

Sample	Raw sequencing reads	Reads after trimming	Reads remaining after host sequence removal	Percentage of reads remaining after host removal (%)
1	259 061 882	253 353 346	4 262 852	1.6%
2	273 131 590	134 050 201	18 371 805	6.7%
3	85 120 864	41 195 609	4 813 354	5.7%
4	110 148 716	53 078 522	3 746 248	3.4%
5	118 989 850	56 597 673	3 454 988	2.9%

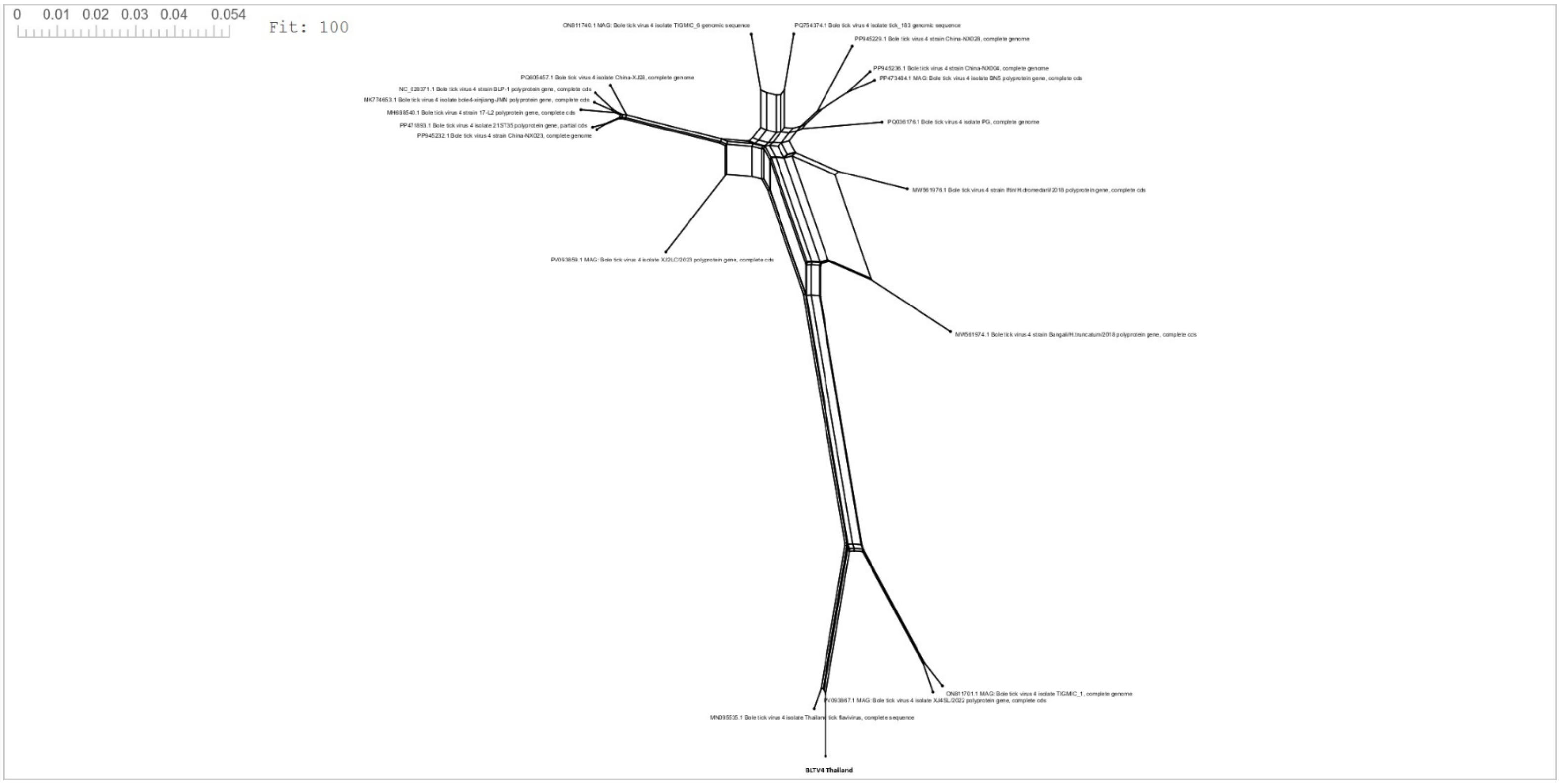

Figure 1. Reticulate network of Bole tick virus 4 (BLTV4) generated in SplitsTree.
